# Prevalence and Morphology of Ossified Caroticoclinoid Ligament: An Updated Systematic Review with Meta-Analysis

**DOI:** 10.3390/diagnostics15040440

**Published:** 2025-02-11

**Authors:** George Triantafyllou, Ioannis Paschopoulos, Sabino Luzzi, George Tsakotos, Panagiotis Papadopoulos-Manolarakis, Renato Galzio, Maria Piagkou

**Affiliations:** 1 Department of Anatomy, Faculty of Health Sciences, School of Medicine, National and Kapodistrian University of Athens, 11527 Athens, Greece; georgerose406@gmail.com (G.T.); johnpascho@gmail.com (I.P.); gtsakotos@gmail.com (G.T.); p.papado89@gmail.com (P.P.-M.); 2 Department of Medicine, Surgery, and Pharmacy, University of Sassari, 07100 Sassari, Italy; sluzzi@uniss.it; 3 Department of Neurosurgery, AOU Sassari, Azienda Ospedaliera Universitaria, Ospedale Civile SS Annunziata, 07100 Sassari, Italy; 4 Department of Neurosurgery, General Hospital of Nikaia-Piraeus, 18454 Athens, Greece; 5 Department of Clinical-Surgical, Diagnostic and Pediatric Sciences, University of Pavia, 27100 Pavia, Italy; renato.galzio@gmail.com

**Keywords:** caroticoclinoid ligament, caroticoclinoid bar, caroticoclinoid foramen, evidence-based anatomy, meta-analysis, variation

## Abstract

**Background**: The caroticoclinoid ligament (CCL) is between the anterior and middle clinoid processes. The ligament can be variably ossified, creating the caroticoclinoid bar or foramen (CCF). When this variant occurs, it encircles the clinoidal segment of the internal carotid artery (ICA) and can cause morphological changes. The present evidence-based systematic review with meta-analysis aims to describe the CCL ossification variability (complete, incomplete, and contact), their pooled prevalence, and the pooled mean of the CCF. **Methods**: The systematic review was performed using four online databases to identify articles that had reported CCF prevalence by its morphology, according to the latest guidelines. The meta-analysis used an R programming software with the “meta” and “metafor” packages. The study protocol was registered in PROSPERO (CRD42024623914). **Results**: The systematic review retrieved a total of 49 studies that had reported on ossified CCL morphological variants. The pooled prevalence of the CCL ossification (irrespective of its morphology) was estimated at 17.47% (95% CI: 14.01–21.23). The most common morphology was the incomplete type, with a pooled prevalence of 10.08% (95% CI: 7.21–13.35). The complete CCF type was calculated at 6.44% (95% CI: 5.30–7.67). The pooled mean diameter of the CCF was 5.00 mm. The geographical distribution, type of study, side, sample size, and sexes did not influence the estimated pooled prevalence. **Conclusions**: The current study systematically reviewed the prevalence and morphology of CCL ossification. Various subgroup analyses were performed to investigate the possible factors affecting it. This variant has adequate clinical significance due to its close relationship with the ICA.

## 1. Introduction

The human skull base exhibits a fascinating typical and variant anatomy. It frequently hides anatomic variants that can significantly complicate all procedures. Knowledge of such variants is essential for clinicians.

**Figure 1:**
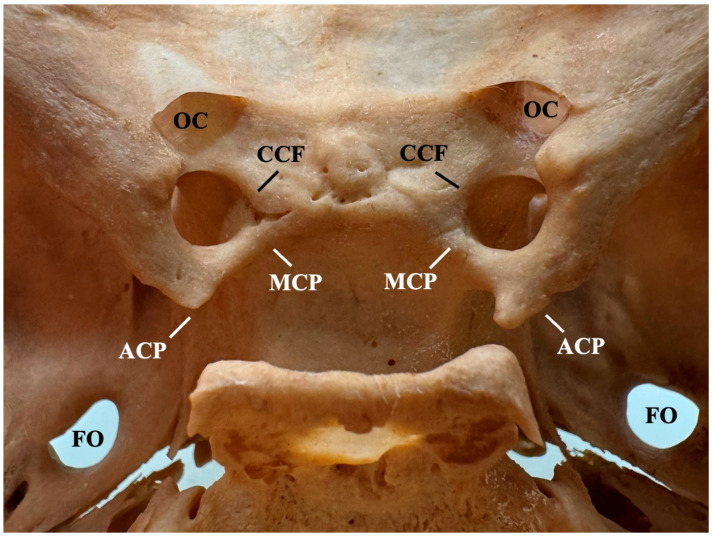
The complete caroticoclinoid foramen (CCF) observed bilaterally in a dry adult skull. ACP—anterior clinoid process, MCP—middle clinoid process, OC—optic canal, FO—foramen ovale.

The sphenoid bone has the following three intracranial clinoid processes: the anterior (ACP), the middle (MCP), and the posterior (PCP) clinoid processes. It is important to highlight that MCP (situated at the body of the sphenoid bone in the anterolateral margin of the sella) is not a constant structure like the ACP and PCP. Each process has significant functions, such as the ACP covering the roof of the cavernous sinus and the para-clinoidal segment of the internal carotid artery (ICA) [1]. The processes are interconnected with ligaments that can be ossified to varying degrees due to the impact of various factors (e.g., age-dependent process) [2]. Specifically, the caroticoclinoid ligament (CCL) is located between the ACP and MCP. Its ossification will result in the formation of a complete or incomplete caroticoclinoid bar (CCB) (Figure 1). The complete ossification of the CCL is more accurately described as the caroticoclinoid foramen (CCF). In addition to the CCL, other ligaments are present in the sella region. The anterior interclinoid ligament can ossify into the homonymous bar between the ACP and PCP. Similarly, the posterior interclinoid ligament can ossify into the posterior interclinoid bar between the MCP and PCP. It is important to highlight, however, that CCB formation is significantly more common than the other sellar bridges [1,2].

The critical ossified structure in the sella region is the CCB, which represents a pathway for the ICA clinoidal segment [3]. Nevertheless, it is located near the cavernous sinus, pituitary gland, and the sphenoid sinus. Bergman’s Comprehensive Encyclopedia of Human Anatomic Variations describes that the CCF can cause morphological changes to the ICA while posing intraoperative neurosurgical difficulties [3]. The CCB pooled prevalence is calculated at 32.1% [4], while the other ossified sellar ligaments (sellar bridges) are less common [2].

The ICA arises from the common carotid artery bifurcation, commonly at the third cervical vertebra level [1]. Several classification systems have been proposed for the ICA segmentation. It is frequently described as seven segments: the cervical (C1), the petrous (C2), the lacerum (C3), the cavernous (C4), the clinoid (C5), the ophthalmic/supraclinoid (C6), and the communicating/terminal (C7) [1]. Thus, the C5 segment is the one implicated in cases of CCF formation. Apart from this variant, other anatomical factors can contribute to ICA compression or stenosis. Vascular tortuosity has been proven to alter the normal flow to the brain, while the ICA has been identified to have a tortuous course in 26.2% of individuals [5]. The proximity of the artery with important bony landmarks has also been proven to be clinically significant. Specifically, the relationship between the ICA and the styloid process (SP) of the temporal bone or the hyoid bone has been systematically studied. Triantafyllou et al. [6] identified that the ICA is closer to the SP when it was elongated, increasing the risk of compression and/or dissection. Nevertheless, Manta et al. [7] observed a wide morphological variability between the carotid arteries and the hyoid bone, recording twelve patterns. This proximity is a cause of compression and atherosclerotic lesions due to the mechanical stress on the arterial wall [7].

We were intrigued by the meta-analysis conducted by Skandalakis et al. [4]; however, we noticed that specific parameters regarding the degree of ossification of the CCL were unclear. Additionally, we identified more relevant articles than those included in the previous systematic review by Skandalakis et al. [4]. Therefore, this systematic review with meta-analysis aims to enhance the current knowledge of the ossified morphological variants of the CCL by further examining its diverse morphology.

## 2. Materials and Methods

The systematic review and meta-analysis were conducted following the PRISMA 2020 guidelines [8] for systematic reviews and the guidelines from the Evidence-Based Anatomy Workgroup [9] for anatomical meta-analysis, consistent with the prior studies [10,11]. Nevertheless, the Anatomical Quality Assurance tool (AQUA) was used to assess the risk of bias within the included studies [12]. This systematic review with meta-analysis has been registered in the PROSPERO online database under identification number CRD42024623914.

Two independent reviewers conducted the literature search and data extraction (GTr, and IP). The results were then compared, and the other authors resolved any discrepancies. Various combinations of the following terms were searched in online databases such as PubMed, Google Scholar, Scopus, and Web of Science until December 2024: “caroticoclinoid bar”, “caroticoclinoid ligament”, “caroticoclinoid foramen”, “variation”, “anatomical study”, “radiological study”, and “imaging study”. Only studies that reported the prevalence of the ossified variants of the CCL were considered eligible for this systematic review. There were no restrictions based on data or language. Exclusion criteria included case reports, animal studies, conference abstracts, letters to the Editor, and studies lacking relevant, sufficient, or complete data. To enhance the literature search, we also performed a hands-on review of the references from eligible articles, along with a search of grey literature and major anatomical journals, including the Annals of Anatomy, Clinical Anatomy, Journal of Anatomy, Anatomical Record, Surgical and Radiological Anatomy, Folia Morphology, European Journal of Anatomy, Morphologie, Anatomical Science International, and Anatomy and Cell Biology. Data were extracted using Microsoft Excel sheets in preparation for statistical analysis.

Statistical meta-analysis was conducted using the open-source R programming language and RStudio software (version 4.3.2, RStudio Team, Boston, MA, USA) using the “meta” and “metafor” packages. The pooled prevalence was calculated using the inverse variance and random effects models. The proportions meta-analysis (prevalence meta-analysis) was conducted using the Freeman–Tukey double arcsine transformation, the DerSimonian–Laird estimator for the between-study variance tau^2^, and the Jackson method for the confidence interval of tau^2^ and tau. Moreover, several subgroup analyses were performed to detect variables affecting the estimated pooled prevalence. The means (mean diameter) meta-analysis was conducted using the untransformed (raw) means, the restricted maximum-likelihood estimator for tau^2^, and the Q-Profile method for confidence interval of tau^2^ and tau. A *p*-value of less than 0.05 was considered statistically significant. Cochran’s Q statistic was used to evaluate the presence of heterogeneity across the studies, and Higgins I^2^ statistic was used to quantify heterogeneity. Cochran’s Q *p*-value < 0.10 was considered significant. Higgins I^2^ values of 0–40% were regarded as not necessary, 30–60% as moderate heterogeneity, 50–90% as substantial heterogeneity, and 75–100% as considerable heterogeneity. To evaluate the presence of a small-study effect (the phenomenon that smaller studies may show different effects than large ones), DOI plots with an LFK index were generated [13].

## 3. Results

**Figure 2:**
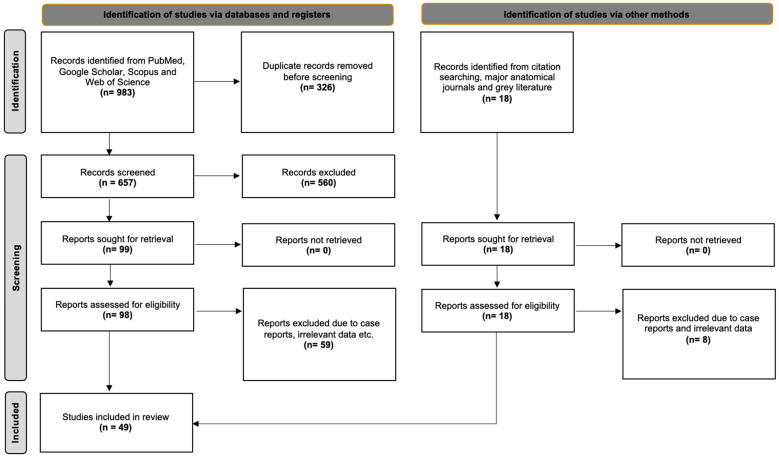
The search analysis flow chart according to PRISMA 2020 guidelines [8].

The database search identified 983 articles exported to Mendeley version 2.10.9 (Elsevier, London, UK). After screening the titles and abstracts to exclude duplicates and irrelevant papers, 99 studies were retrieved for the full-text review. Ultimately, 39 studies met the criteria for inclusion in this systematic review. Additionally, we identified 10 more studies through a secondary investigation, which included references, grey literature, and hands-on searches of anatomical journals. Consequently, 49 studies were included in our systematic review with meta-analysis. Figure 2 presents a flow diagram of our search process, following the PRISMA 2020 guidelines.

Forty-nine (49) studies were included, comprising 21,349 skull sides. Of these, thirty-eight (38) studies were osteological, while eleven (11) utilized imaging techniques. The average number of skull sides per article was 426.98. The distribution of the articles by population was as follows: twenty-eight (28) articles focused on the Asian population, nine (9) on the American population, eight (8) on the European population, and three (3) on the African population. The characteristics of the included studies are summarized in Table 1.

Typical ACP-MCP regional anatomy was observed in 82.53% of cases (95% CI: 78.86–85.76). The overall pooled prevalence of CCL ossification, regardless of its morphology, was 17.47% (95% CI: 14.01–21.23). This variant was noted to occur unilaterally in 11.27% of cases (95% CI: 8.99–13.77) and bilaterally in 9.87% (95% CI: 6.82–13.37), with no laterality impact (*p* = 0.5013).

The CCB incomplete type was estimated to have a pooled prevalence of 10.08% (95% CI: 7.21–13.35), while the CCB complete type had a pooled prevalence of 6.44% (95% CI: 5.30–7.67). The rarest type identified was the CCB contact type, with a pooled prevalence of 1.13% (95% CI: 0.56–1.84%).

Finally, the CCF diameter was estimated to have a pooled mean of 5.00 mm (95% CI: 4.69–5.13), with no significant difference between the sides (*p* = 0.8050).

Table 2 summarizes a subgroup analysis of geographical distribution, study type, and sample size. The nationality subgroup retrieved a statistically significant difference for the CCB complete type (*p* = 0.0044) and incomplete type (*p* = 0.0010). The type of study (osteological or imaging) exhibited a statistically significant result for the CCB contact type (*p* = 0.0154).

Additionally, the analysis of sides and sexes is presented in Table 3. The DOI plots for each pooled prevalence showed an LFK index ranging from −1.33 to −2.91, indicating minor to major asymmetry. This suggests that a small study effect should be considered for the evaluated parameters.

## 4. Discussion

The current meta-analysis aimed to investigate the pooled prevalence of the variable morphology of the CCL ossification types and included several subgroup analyses. We meticulously examined 49 studies that analyzed a remarkable total of 21,349 skull sides. We reported an overall pooled prevalence of 17.47%, stratified solely by skull side, as most studies did not provide data stratified by skull. We calculated the bilateral occurrence in 9.87% of skulls. Furthermore, we presented pooled prevalence data for the possible morphology of the ossified CCB, considering variations based on sides and sexes. In a previous meta-analysis of the CCF by Skandalakis et al. [4], 26 studies with a combined sample of 7521 skulls were examined. They reported a pooled prevalence of 32.6% when stratified by skull and 23.6% when stratified by skull side. Additionally, they identified a bilateral occurrence rate of 41.1%. Regarding the CCF morphology, they found that the complete or contact type was present in 46% of cases. Our meta-analysis study improves the understanding of the ossified morphological variants of the CCL by examining all ossification patterns based on side and sexes.

The development of the sphenoid involves the endochondral ossification centers observed in the orbitosphenoid cartilage around the ninth developmental week. However, some studies suggest that these ossification centers may appear closer to the twelfth week. The development of the main ossification centers of the presphenoid varies among individuals [62]. Notably, differences in size can be observed, as well as size discrepancies between the left and right sides [62]. Additionally, posterior accessory centers may emerge at the posterolateral ends of the primary presphenoid ossification centers. Kodama [62] reported that these posterior accessory centers were found in only 2 out of 15 skulls (13.33%) from embryos measuring 29.0 and 31.5 cm long. In both instances, the accessory centers appeared bilaterally. The author posits that presphenoid asymmetrical development may affect CCF development. Moreover, the inconsistent appearance of posterior accessory ossification centers might play a role in CCF formation, potentially contributing to MCP growth. Zdilla et al. [52] showed that the CCF may begin to develop in the fourth fetal month before the orbitosphenoid bones merge with the presphenoid portion of the sphenoid.

Extensive research has been carried out on the prevalence and ossified morphology of the CCL, which is summarized in the current review. Speculations have arisen about the potential nationality distribution of CCF formation [17,19]. In the present meta-analysis, the continent of origin did not significantly influence the overall pooled prevalence of CCL ossification (Table 2). A statistically significant finding emerged regarding the pooled prevalence of the complete and incomplete CCB types. Studies from Africa indicated the highest prevalence of the complete type, while European studies reported the highest prevalence of the incomplete type. It is important to interpret these subgroup analysis results cautiously, as a minimum of four studies per parameter was unmet [63]. Additionally, CCL ossification may not be influenced by age [39], which distinguishes it from the ossification seen in other ligaments. Several studies found no significant association with age [2,61], and CCB presence in fetal skulls supports this theory [52]. Zdilla et al. [52] reported that the prevalence of CCF among newborns was 45.8% (11 out of 24 sides) when assessed laterally and 50% (6 out of 12 crania) when examined by the skull, which is higher than the prevalence typically observed in adults. Furthermore, the type of study (osteological or imaging) did not retrieve a statistically significant result, except for the CCF contact type. However, this should be taken into careful consideration because the subgroup analysis did not satisfy the minimum of four studies per subgroup. The subgroup analysis based on the study of type proves that imaging techniques, such as computed tomography scans, are adequate to identify this variation.

The relationship between the skull base variants and the ICA remains largely unclear. It is widely believed that a CCB may induce morphological (regional) changes in the ICA [3]. In this meta-analysis, the pooled mean diameter of the CCF was 5.00 mm. However, further clinical assessment was not possible due to the lack of data regarding ICA morphometry. This interpretation was accurately demonstrated by Paschopoulos et al. in their study, where they emphasized the significance of calculating the CCF diameter while considering the ICA diameter [61]. In a survey by Ozdogmus et al. [17], researchers examined donor heads that had preserved soft tissue and assessed the ICA diameter. Their findings indicated a positive correlation between the ICA diameter and the right CCF. Paschopoulos et al. [61] investigated the CCF presence and morphology in a sample of 260 dried skulls and computed tomography (CT) scans. They subsequently compared these findings with 30 computed tomography angiograms (CTAs) of individuals lacking a CCF, measuring the ICA diameter at the ACP and MCP areas, which ranged from 4.0 to 5.0 mm. They identified three morphological stenosis patterns of the CCF based on the ICA diameter, suggesting that a CCF with a diameter of less than 4.0 mm poses a high risk of compression, which was present in 16.8% of their sample. Moreover, the study indicated that female specimens had a significantly higher prevalence of CCF associated with a “high risk” of compression [61]. Neurosurgeons often perform clinoidectomies of the ACP and MCP to access the cavernous sinus and the paraclinoidal and parasellar regions [4]. The presence of a CCF can displace the ICA, which increases the risk of iatrogenic injury. Additionally, there is a potential risk of intraoperative fracture of the carotid canal [4]. Nevertheless, Ota et al. evaluated the CCF occurrence in patients with paraclinoid aneurysms using a multidetector CT and determined it to be present in 16.6% of the patients [33].

The present study has a few limitations. Firstly, several studies showed a “high” risk of bias according to the AQUA tool [9]. Most pooled prevalence estimates displayed significant heterogeneity and varying degrees of asymmetry in their DOI plots. However, anatomical meta-analyses commonly observe these findings [12]. Lastly, some subgroup analyses did not include the minimum of four studies per parameter, as recommended [63]. Further research should focus on examining the presence of the CCF, along with their morphology and morphometry. Studies investigating concomitantly the diameters of the CCF and ICA clinoid segment, similar to the method proposed by Paschopoulos et al. [61], will enhance our knowledge about their relationship.

## 5. Conclusions

The present systematic review, bolstered by a meta-analysis, has enhanced our understanding of the morphological variability in CCL ossification. The overall pooled prevalence of CCL ossification, regardless of its morphology, is 17.47%. Most commonly, it is identified unilaterally in its incomplete form and with a pooled mean diameter of 5.00 mm. Although the presence of this variant has been thoroughly examined in the existing literature, its relationship with the ICA has been infrequently reported. Future research into this relationship will provide valuable insights for the neurosurgeons working in this area.

## Figures and Tables

**Table 1:** The characteristics of the included studies and their risk of bias according to the AQUA tool [12].

Study	Year	Population	Type of Study	Skulls (N)	Age Group	Risk of Bias
Keyes [14]	1935	America	Osteological	2187	Infants- Adults	Low
Inoue et al. [15]	1990	America	Osteological	50	NR	High
Lee et al. [16]	1997	Asia	Osteological	73	Adults	High
Ozdogmus et al. [17]	2003	Asia	Osteological	50	Adults	Low
Anukusampan et al. [18]	2004	Asia	Imaging	200	Adults	Low
Eftrhymiou et al. [19]	2004	Asia	Osteological	76	Adults	Low
Erturk et al. [20]	2004	Europe	Osteological	171	Adults	High
Gupta et al. [21]	2005	Asia	Osteological	70	NR	High
Archana et al. [22]	2010	Asia	Osteological	250	Adults	High
Desai et al. [23]	2010	Asia	Osteological	100	NR	High
Boyan et al. [24]	2011	Asia	Osteological	34	Adults	High
Freire et al. [25]	2011	America	Osteological	80	Adults	Low
Aggarwal et al. [26]	2012	Asia	Osteological	67	Adults	High
Fernandez-Miranda et al. [27]	2012	America	Osteological and Imaging	300	NR	High
Kapur et al. [28]	2012	Europe	Osteological	200	Adults	Low
Kinjiya [29]	2012	Asia	Osteological	200	NR	High
Shaikh et al. [30]	2012	Asia	Osteological	200	NR	High
Peris-Celda et al. [31]	2013	America	Osteological	270	NR	High
Dagtekin et al. [32]	2014	Asia	Osteological	40	NR	High
Ota et al. [33]	2014	Asia	Imaging	77	Adults	Low
Brahmbhatt et al. [34]	2015	Asia	Osteological	50	Adults	High
Suprasanna et al. [35]	2015	Asia	Imaging	95	Adults	Low
Miller et al. [36]	2016	America	Imaging	150	Adults	Low
Souza et al. [37]	2016	Asia	Osteological	27	NR	High
Bansode et al. [38]	2017	Asia	Osteological	35	Adults	High
Gibelli et al. [39]	2017	Europe	Imaging	300	Adults	Low
Jha et al. [40]	2017	Asia	Osteological	108	Adults	Low
Muthukumar et al. [41]	2017	Asia	Osteological	100	NR	High
Suprasanna et al. [42]	2017	Asia	Imaging	54	Adults	Low
Kumar et al. [43]	2018	Asia	Osteological	50	Adults	High
Natsis et al. [44]	2018	Europe	Osteological	123	Adults	Low
Purohit et al. [45]	2018	Asia	Osteological	200	Adults	High
Savikumar et al. [46]	2018	Asia	Osteological	300	NR	High
Sharma et al. [47]	2018	America	Osteological	2726	Adults	Low
Sibuor et al. [48]	2018	Africa	Osteological	51	NR	High
Gulbes et al. [49]	2019	Asia	Imaging	100	Children and Adults	Low
JaiSankar et al. [50]	2019	Asia	Osteological	50	Adults	Low
Touska et al. [51]	2019	Europe	Imaging	240	Children and Adults	Low
Zdilla et al. [52]	2019	America	Osteological	100	Fetuses and Adults	High
Elbadawi et al. [53]	2020	Africa	Osteological	30	Adults	High
Pires et al. [54]	2020	America	Osteological	365	NR	High
Prathiba et al. [55]	2020	Asia	Osteological	100	Adults	High
de Oliveira et al. [56]	2021	America	Osteological	38	NR	High
Priya et al. [57]	2021	Asia	Osteological	50	Adults	High
Nikolova et al. [58]	2023	Europe	Imaging	315	Children and Adults	Low
Piagkou et al. [2]	2023	Europe	Osteological	156	Adults	Low
Rao et al. [59]	2023	Asia	Osteological	100	Adults	High
Magcaba et al. [60]	2024	Africa	Osteological	32	NR	High
Paschopoulos et al. [61]	2025	Europe	Imaging	260	Adults	Low

**Table 2:** The presence of caroticoclinoid ligament (CCL) ossified variants according to nationality, type of study, and sample size. Statistically significant results are presented with asterisk.

Parameters	Type of the CCL Ossification			
	Total (All Types)	Complete Type	Incomplete Type	Contact Type
By the geographic area				
Asia	16.30%	5.58%	10.85%	1.37%
Europe	24.35%	9.34%	12.26%	2.17%
Africa	11.75%	13.23%	0.00%	0.00%
America	16.98%	5.36%	8.18%	0.88%
*p*-value	0.1230	0.0044 *	0.0010 *	0.1715
By the study’s type				
Osteological	17.74%	6.29%	11.01%	0.73%
Imaging	16.60%	7.07%	7.35%	3.11%
*p*-value	0.7551	0.6160	0.3803	0.0154 *
By the number of the examined sample				
Sample > 100 patients	18.30%	6.05%	10.85%	1.04%
Sample < 100 patients	17.18%	8.42%	8.04%	1.92%
*p*-value	0.7546	0.2972	0.5568	0.4593

**Table 3:** The presence of caroticoclinoid ligament (CCL) ossified variants according to sides and sexes.

Parameters	Type of CCL Ossification			
	Total (All Types)	Complete Type	Incomplete Type	Contact Type
By the sex				
Female	22.69%	9.08%	18.90%	0.90%
Male	27.74%	7.82%	16.79%	0.60%
*p*-value	0.3126	0.6722	0.7625	0.8037
By the side				
Left	16.99%	6.17%	8.73%	0.36%
Right	19.65%	7.33%	9.53%	0.35%
*p*-value	0.3425	0.3478	0.7307	0.9646

## Data Availability

All the data are available upon reasonable request to the corresponding author (Professor Maria Piagkou: mapian@med.uoa.gr).

## Abbreviations

The following abbreviations are used in this manuscript:

CCL	Caroticoclinoid ligament
CCB	Caroticoclinoid bar
CCF	Caroticoclinoid foramen
ACP	Anterior clinoid process
MCP	Middle clinoid process
PCP	Posterior clinoid process
ICA	Internal carotid artery
SP	Styloid process
AQUA	Anatomical Quality Assurance Tool
